# Peroxynitrite Mediates Diabetes-Induced Endothelial Dysfunction: Possible Role of Rho Kinase Activation

**DOI:** 10.1155/2010/247861

**Published:** 2010-11-01

**Authors:** Azza B. El-Remessy, Huda E. Tawfik, Suraporn Matragoon, Bindu Pillai, Ruth B. Caldwell, R. William Caldwell

**Affiliations:** ^1^Program in Clinical and Experimental Therapeutics, College of Pharmacy, The University of Georgia, 1120 15th Street, HM-1200, Augusta, GA 30912, USA; ^2^Department of Pharmacology & Toxicology and Vascular Biology Center, Medical College of Georgia, 1120 15th Street, HM-1200, Augusta, GA 30912, USA; ^3^Vascular Biology Center, Medical College of Georgia, GA 30912, USA; ^4^Charlie Norwood VA Medical Center, Augusta, GA 30912, USA; ^5^King Saud University, Riyadh 11451, Saudi Arabia

## Abstract

Endothelial dysfunction is characterized by reduced bioavailability of NO due to its inactivation to form peroxynitrite or reduced expression of eNOS. Here, we examine the causal role of peroxynitrite in mediating diabetes-induced endothelial dysfunction. Diabetes was induced by STZ-injection, and rats received the peroxynitrite decomposition catalyst (FeTTPs, 15 mg/Kg/day) for 4 weeks. Vasorelaxation to acetylcholine, oxidative-stress markers, RhoA activity, and eNOS expression were determined. Diabetic coronary arteries showed significant reduction in ACh-mediated maximal relaxation compared to controls. Diabetic vessels showed also significant increases in lipid-peroxides, nitrotyrosine, and active RhoA and 50% reduction in eNOS mRNA expression. Treatment of diabetic animals with FeTTPS blocked these effects. Studies in aortic endothelial cells show that high glucose or peroxynitrite increases the active RhoA kinase levels and decreases eNOS expression and NO levels, which were reversed with blocking peroxynitrite or Rho kinase. Together, peroxynitrite can suppress eNOS expression via activation of RhoA and hence cause vascular dysfunction.

## 1. Introduction

Diabetes mellitus predisposes patients to premature atherosclerotic coronary artery disease, the leading cause of morbidity and mortality among patients with diabetes [1, 2]. The vascular endothelium is a target of the diabetic milieu, and endothelial dysfunction has been thought to play a central role in diabetic vascular diseases (reviewed in [3]). Diabetes-induced endothelial dysfunction is characterized by reduced bioavailability of nitric oxide (NO) in the vessel wall. NO, a major regulator of vascular tone, is produced by the activity of endothelial NO synthase (eNOS). Diminished capacity of eNOS to generate NO has been demonstrated when endothelial cells were exposed to elevated glucose levels either *in vitro *or *in vivo* [4–7]. In response to hyperglycemia, an imbalance between increased production of superoxide anion (O_2_^.−^) and NO drives the formation of peroxynitrite (ONOO^−^) within the vascular wall [8]. Peroxynitrite can oxidize the NOS cofactor tetrahydrobiopterin and also reduce cellular transport of L-arginine, eNOS substrate for NO production [9]. These events uncouple the enzyme, which then preferentially increases O_2_^.−^ production over NO production leading to a vicious cycle of peroxynitrite formation and further inactivation of NO [4, 10]. 

Recent studies raised the possibility that diabetes-impaired NO bioavailability could be caused by reduced expression of eNOS, in addition to the known role of O_2_^.−^ to inactivate NO [11–13]. Accumulating evidence indicates that expression of eNOS is regulated by the RhoA/ROCK pathway [14, 15]. The small GTP-binding protein RhoA GTPase and its downstream target, the Rho-associated kinase (ROCK), are implicated in a variety of physiological functions of endothelial cells including cell adhesion, motility, migration, and contraction [16]. Inhibition of the RhoA/ROCK pathway indirectly by statins or directly by ROCK inhibitors or dominant-negative mutant of RhoA has been shown to increase eNOS expression [17–19]. Our previous studies demonstrated significant upregulation of the active RhoA that positively correlated with increases in peroxynitrite as well as vascular permeability and impaired vasorelaxation in models of experimental diabetes [20, 21]. However, the causal role of peroxynitrite in mediating diabetes-induced endothelial dysfunction and the potential vascular protective effects of the peroxynitrite decomposition catalyst, FeTTPs, have not been elucidated. Our goal is to examine the effects of decomposing peroxynitrite and to explore the possible role of RhoA in modulating eNOS expression in rat vessels and cultured aortic endothelial cells in response to diabetes and hyperglycemia, respectively.

## 2. Material and Methods

### 2.1. Animal Preparation

All procedures with animals were performed according to the “Principles of Laboratory Animal Care” (NIH publication no. 85023, revised 1985) and the guidelines of the VA Medical Center and Medical College of Georgia Animal Care and Use Committees. Male Sprague-Dawley rats (~250 g body weight) were randomly assigned to: control, treated-control, diabetic, or treated-diabetic groups. Three sets of animals were prepared (totaling 62 rats) to study the effects of 4 weeks of experimental diabetes. Diabetes was induced by intravenous tail-vein injection of streptozotosin (65 mg/kg). After 48 hours, diabetic status was determined by urine detection of glucose. Diabetes was confirmed with blood-glucose levels >350 mg/dl, which correspond to average blood levels of poorly-controlled diabetic patients. The animals were treated with the peroxynitrite decomposition catalyst, FeTPPs [5,10,15,20-tetrakis (4-sulfonatophenyl) porphyrinato iron (III)] (Calbiochem, CA), via intraperitoneal, (IP) injections at 15 mg/kg. FeTPPs exhibits minimal SOD mimetic activity, does not complex with nitric oxide, and catalytically isomerizes peroxynitrite to nitrate. After 4 weeks of diabetes, animals were sacrificed, and vessels were isolated for analyses.

### 2.2. Preparation of Rat Coronary Arteries

Rats were anesthetized with intraperitoneal injection of ketamine HCl (20 mg/kg) and xylazine (4 mg/kg). A thoracotomy was performed; heart and thoracic aorta were quickly excised and placed in ice-cold oxygenated Krebs-Henseleit buffer. After the right ventricle and anterior wall of the left ventricle were removed under a stereomicroscope, intramyocardial second-order branches of the septal arteries were dissected from the septum facing the right ventricular cavity. Arterial segments (~2 mm long) were gently excised, transferred to the chamber of a small vessel myograph (Danish Myo Technology) containing 5 ml buffer, and mounted on tungsten wires (diameter 40 *μ*m). The arteries were allowed to equilibrate in oxygenated (95% O2–5% CO2) Krebs-Henseleit buffer. The composition of the buffer is (in mM) 118.3 NaCl, 4.7 KCl, 1.2 MgSO4, 1.2 KH2PO4, 25 NaHCO3, 2.5 CaCl2, and 11.0 glucose. Vessels were allowed to stabilize for 30 min in the Krebs-Henseleit buffer under zero tension, during that time the buffer solution was changed at 10 min intervals. The vessels were then radially stretched to their optimal lumen diameter for active tension development, that is, to an internal circumference equal to 90% of that achieved in vessels exposed to a passive tension equivalent to a transmural pressure of 100 mmHg. Isometric force was recorded on a computer by use of Chart v 5 software and a MacLab/4e data acquisition system (AD Instruments).

### 2.3. Protocol for Acetylcholine Dose-Response Curves

After the equilibration period, the responsiveness of each individual artery was confirmed by its successive vasoconstrictive response to a submaximally concentration of KCl (125 mM). The integrity of the vascular endothelium was tested pharmacologically by acetylcholine-induced relaxation of arteries that had been precontracted with U46619 (thromboxane A_2_ receptor agonist). Tissues that did not elicit a reproducible and stable contraction with U46619 (1 *μ*M) and relaxed >40% in response to 10 *μ*M acetylcholine were discarded from the study. Preparations were then washed three times with K-H buffer and allowed to relax fully for 30 min before the experimental protocol began. Coronary arteries were then again precontracted with U46619 at a submaximal dose of 1 *μ*M (*E*_max_, 10 *μ*M). After reaching a plateau of contraction, cumulative concentration-response curves to acetylcholine (ACh, 0.1 nM–100 *μ*M) were obtained to evaluate endothelium dependent vasorelaxation. The concentration in the chamber was increased in 1-log steps. In all cases, ACh was added to yield the next higher concentration only when the response to the earlier dose reached a steady state. The vasorelaxant responses are expressed as percent decrease from U-46619-induced contraction; that is, the amount of contraction produced by 1 *μ*M U-46619 in each vessel from its initial resting tension was considered to be 100%.

### 2.4. Determination of Nitrotyrosine

Slot-blot analysis was used to verify the efficacy of FeTPPs of blocking tyrosine nitration in treated animals. As described previously [22, 23], 30 *μ*g of aortic homogenate from rat samples was immobilized onto a nitrocellulose membrane. After blocking, membranes were reacted with antibody against nitrotyrosine (Calbiochem), followed by secondary antibody, and enhanced chemiluminescence (GE Health Care), and the optical density of various samples was compared with that of controls.

### 2.5. Determination of Lipid Peroxides

The assay was performed on aortic lysates as described before [22]. Briefly, aortic lysate is reacted with 20% acetic acid, 8% SDS, and thiobarbituric acid at 95°C for 60 min, and the reaction was cooled down on ice. The samples were centrifuged, and the supernatant was extracted with n-butanol and pyridine (15 : 1, resp.), and the absorbance of the organic solvent layer was measured at 532 nm. The results were compared with an external standard (Tetramethoxypropane). The Bradford assay (Bio-Rad, Hercules, CA) was performed to determine the protein concentration of the retinal lysate. Lipid peroxide level was expressed in nmol MDA/mg total protein.

### 2.6. Preparation of mRNA and eNOS Expression

Aortic endothelium mRNA was prepared according to manufacturer's instructions using a Promega kit. The One-step qRT-PCR Invitrogen kit was used to amplify 10 ng of mRNA from each sample. A pair of rat-specific eNOS primers was synthesized to amplify a 123-bp DNA fragment forward-primer 5′-TGACCCTCACCGATACAACA and reverse-primer 5′-AACGTGGCTGTGCTGTACAG. The Rat 18-S was used as an internal marker for each sample. Forward-primer: CGCGGTTCTATTTTGTTGGT and the reverse-primer: AGTCGGCATCGTTTATGGTC. Quantitative PCR was performed using a Realplex Mastercycler (Eppendorf, NY). Expression of eNOS was normalized to 18S level in each sample and expressed as relative expression to untreated controls.

### 2.7. Cell Culture Studies

 Primary cultures of bovine aortic endothelial (BAE) cells were prepared as described previously in [24]. Cells from *passages 4–8* were used in all experiments. Cells were maintained in M199 supplemented with 10% FBS, 10% CS-C complete medium, 2 mM glutamine, 100 U/ml penicillin, and 100 *μ*g/ml streptomycin at 37°C in a humidified CO_2_ incubator. Cells were incubated in 5.5 mM (normal) or 25 mM (high) D-glucose for 3 days with and without FeTPPs (2.5 *μ*M) or treated with exogenous peroxynitrite 100 *μ*M for 18 hours.

### 2.8. Determination of Oxidative Stress Using DCF Assay

 The oxidation of 2,7-dihydrodichloroflurescein diacetate (DHCF) from Molecular Probes (Eugene, OR) was used. DCF is the oxidation product of DHCF and is widely used as a general marker of cellular oxidation by hydroxyl radicals, hydrogen peroxide, and peroxynitrite. The cultures were treated with DHCF (5 *μ*M) for 1 h in the presence or absence of the peroxynitrite decomposition catalyst FeTPPs (2.5 *μ*M), and the fluorescence was measured at excitation 495 and emission 520.

### 2.9. Western Blot

For analysis of eNOS, aortic endothelial cells were homogenized in a modified RIPA buffer [20 mM Tris-HCl (pH 7.4), 2.5 mM ethylenediamine tetraacetic acid, 50 mM NaF, 10 mM Na_4_P_2_O_7_, 1% Triton X-100, 0.1% sodium dodecyl sulfate, 1% sodium deoxycholate, and 1 mM phenylmethyl sulfonyl fluoride]. Total protein concentrations were measured using Bio-Rad protein assay. Protein samples (40 *μ*g) were separated by 7.5% sodium dodecyl sulfate-polyacrylamide gel electrophoresis, transferred to nitrocellulose membrane, and probed with anti- eNOS (Cell Signaling) and actin (Sigma) followed by secondary antibody and enhanced chemiluminescence (GE Health Care). The films were subsequently scanned, and band intensity was quantified using densitometry software (alphEaseFC) and expressed as relative optical density (ROD).

### 2.10. RhoA Activity

RhoA activity was assessed by pull-down assay as previously described in [20]. In brief, aortas were homogenized in assay buffer (50 mM Tris, pH 7.2; 1% Triton X-100; 0.5% sodium deoxycholate; 0.1% SDS; 500 mM NaCl; 10 mM MgCl_2_ and protease inhibitors). Next, 200 *μ*g of homogenate was incubated with 10 *μ*g of agarose (GST)-TRBD beads at 4°C for 60 min. The beads were washed four times with the assay buffer. Bound Rho proteins were then detected by Western blot by using a monoclonal antibody against RhoA (Millipore).

### 2.11. Nitric Oxide Assay

For analysis of NO formation *in vitro*, we used an NO analyzer (Sievers). BAEC in 24-well plates were incubated in 5.5 mM (normal) or 25 mM (high) D-glucose with and without simvastatin (1 *μ*M). After 3 days, media were processed for the measurement of nitrite (NO^−^_2_), the stable breakdown product of NO in aqueous solution, by NO-specific analyzer. Media were deproteinized, and samples containing NO^−^_2_ were refluxed in glacial acetic acid containing sodium iodide. Under these conditions, NO^−^_2_ was quantitatively reduced to NO, which was quantified by a chemiluminescence detector after reaction with ozone in the NO analyzer.

### 2.12. Data Analysis

All values are shown as mean ± SEM. Maximal relaxation (*E*_max_\) and half-maximal effective dose (EC_50_) were calculated from individual dose-response curves. EC_50_ values were derived using Graph-Pad Prism. Differences among experimental groups were evaluated by ANOVA, and the significance of differences between groups was assessed by the post hoc test (Fisher's PLSD) when indicated. Significance was defined as *P* < .05.

## 3. Results

### 3.1. FeTPPs Did Not Alter Body Weight or Blood Glucose Level

As shown before, treatment with FeTPPs had no effect on body weight or blood glucose levels in diabetic rats [22]. STZ-injected animals had significant increases of blood glucose level (480 ± 24 mg/dL) compared to control rats (185 ± 14 mg/dL). Treatment with FeTPPs had no significant effect on the blood glucose levels in diabetic rats (567 ± 29 mg/dL) or in treated controls (179 ± 12 mg/dL).

### 3.2. FeTPPs Improves Diabetes-Impaired Coronary Endothelial-Dependent Vasorelaxation

**Figure 1:**
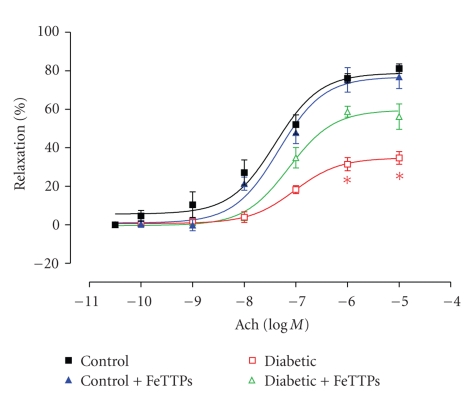
FeTPPs improves diabetes-impaired coronary endothelial-dependent vasorelaxation. Concentration-response curves for the effect of ACh for coronary arteries from control, diabetic, FeTPPs-treated control, and FeTPPs-treated diabetic rats at 4 weeks (*n* = 6 in each group). ACh *E*_max_ value from diabetic vessels was significantly lower than control. Cotreatment with FeTPPs improved *E*_max_ value from diabetic vessels but did not affect treated controls. Values are expressed as means ± SEM, *P < .05 versus control.

We studied the effect of diabetes on coronary endothelial-dependent vasorelaxation by performing ACh concentration-response curves with coronary arteries from diabetic and age-matched control rats. As shown in Figure 1, ACh produced concentration-dependent vasorelaxation in coronary arteries from all groups. ACh produced maximal relaxation (*E*_max_\) of 81% ± 2.5 in coronary arteries from control rats with an EC_50_ value of 42 ± 0.15 nM. However, coronary arteries from 4 weeks diabetic rats exhibited decreased *E*_max_ to ACh (34.7%  ± 3.2) and a shift in the concentration response curve to the right with an EC_50_ value of 94 ± 0.2 nM. Treatment with FeTPPs significantly improved *E*_max_ relaxation to ACh to 58.8% ± 2.8, and EC_50_ was decreased to 70 ± 1.3 nM (Figure 1). Treatment of controls with FeTPPs did not alter the response to ACh. Coronary arteries from various groups relaxed in a similar fashion to the NO donor SNP as shown in our previous studies [6, 20]. These data confirmed that, in our model, diabetes impairs endothelial-dependent-relaxation only and has no effect on smooth muscle relaxation.

### 3.3. FeTPPs Blocked Diabetes-Induced Oxidative Stress and Nitrotyrosine Formation in Rat Vessels

**Figure 2:**
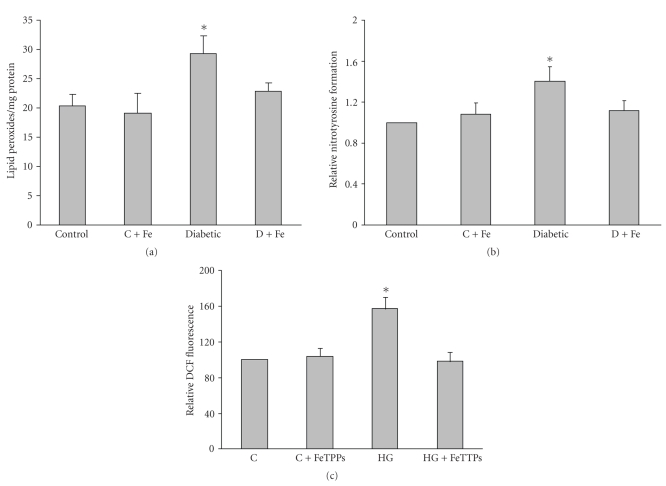
FeTPPs blocked diabetes-induced oxidative stress and nitrotyrosine formation in rat vessels. Effect of diabetes on oxidative and nitrative stress in rat aortic homogenate was determined by lipid peroxides using MDA assay (a) and nitrotyrosine immunoreactivity using slot blot (b). Both lipid peroxides and nitrotyrosine were significantly increased in diabetic vessels. Treatment with FeTTPS blocked these effects in diabetic (D + Fe) but not in treated controls (C + Fe). Values are expressed as means ± SEM, (*n* = 6 in each group,  *P < .05 versus control). (c). Effect of FeTPPs on DCF fluorescence. Bovine aortic endothelial cells (BAEC) were incubated with normal (NG, 5 mM) and high glucose (HG, 25 mM) for 3 days (*n* = 5 in each group). High glucose-increased ROS formation, which was prevented by cotreatment with FeTPPs. Values are expressed as means ± SEM, *P < .05 versus control.

Diabetes-induced oxidative stress was analyzed by measuring lipid peroxides and nitrotyrosine in aorta homogenate from various groups. The results showed that diabetes causes significant increases (1.5 fold) in lipid peroxides as well as tyrosine nitration (1.4 fold) compared to control animals. Treatment of diabetic rats with FeTPPs (15 mg/Kg/day) significantly reduced lipid peroxidation and nitrotyrosine formation in diabetic vessels to control levels (Figures 2(a) and 2(b)).

### 3.4. FeTPPs Blocked High-Glucose-Increased ROS Formation in BAEC

In order to further define the impact of the diabetic milieu on the vascular endothelium, we determined the effect of high glucose on ROS produced by bovine aortic endothelial cells (BAEC) using the oxidized dichlorofluorescein fluorescence (DCF), a commonly used marker for both oxidative and nitrative stress. The results showed that incubation with 25 mM glucose for 3 days caused an increase in ROS formation of ~58% above controls. Concurrent treatment with the specific peroxynitrite decomposition catalyst FeTPPs (2.5 *μ*M) completely blocked high glucose-induced ROS formation (Figure 2(c)). These results suggest that peroxynitrite is the major free radical produced by high glucose treatment.

### 3.5. FeTPPs Restored Nitric Oxide Production in Diabetic Vessels

**Figure 3:**
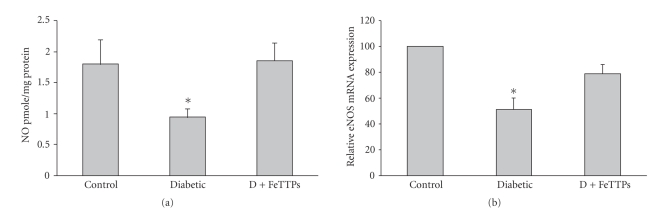
FeTPPs restored nitric oxide production and eNOS expression in diabetic rat vessels. (a) Diabetes significantly reduced NO formation in rat aortic homogenate as measured by NO analyzer. Cotreatment of diabetic animals with FeTPPs restored NO back to normal levels (*n* = 6 in each group). Values are expressed as means ± SEM, *P < .05 versus control. (b) Real-time PCR of eNOS expression from aortic endothelial homogenate showed that diabetes significantly decreased eNOS expression (~50%) of control level, and FeTPPs restored eNOS expression to 87% of basal level (*n* = 5 in each group). Values are expressed as means ± SEM, *P < .05 versus control.

In order to test whether the increased oxidative stress in diabetic rats reduces NO availability, we determined NO formation using a NO analyzer. As shown in Figure 3(a), diabetes significantly reduced NO production by 50% below control level. Cotreatment of diabetic animals with FeTPPs (15 mg/Kg/day) prevented the reduction in NO production to control levels.

### 3.6. FeTPPs Restored eNOS Expression in Diabetic Vessels

In order to determine whether FeTPPs improves NO production through altering eNOS expression, we measured eNOS mRNA levels from isolated rat aortic endothelium from various groups. Diabetes significantly reduced eNOS expression (54% of basal level) in aortic endothelium compared to controls (Figure 3(b)). Treatment of diabetic animals with FeTPPs restored eNOS back to 87% of basal level. These data suggest that FeTPPs improved endothelial dysfunction via a mechanism that involves an action of inhibiting the reduction of eNOS expression.

### 3.7. FeTPPs Inhibited Activation of RhoA in Diabetic Vessels and High-Glucose-Treated BAEC

**Figure 4:**
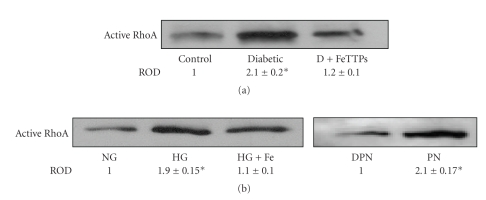
FeTPPs inhibited diabetes-induced RhoA activation in rat vessels and BAEC. (a) Pull-down assay showed significant increases in active RhoA in diabetic aortic homogenate compared to that of controls that were blocked by cotreatment with FeTPPs (*n* = 6). Values are expressed as means ± SEM, *P < .05 versus control. (b) Bovine aortic endothelial cells (BAECs) were incubated with normal (NG, 5 mM) and high glucose (HG, 25 mM) for 3 days or exogenous peroxynitrite (PN, 100 *μ*M) for 18 hours (*n* = 4 in each group). High glucose significantly increased active RhoA, which was prevented by cotreatment with FeTPPs (Fe, 2.5 *μ*M). Peroxynitrite exerted similar effects to high-glucose in increasing active RhoA (~2-fold) compared to decomposed peroxynitrite (DPN). Values are expressed as means ± SEM, *P < .05 versus control.

Activation of small GTPases such as RhoA has been shown to modulate eNOS expression at the mRNA level. Therefore, we measured the levels of active RhoA in aortic endothelial homogenate from various groups. The results showed that diabetes causes significant activation of RhoA (2-fold) compared to controls that blocked by treatment with FeTPPs (Figure 4(a)). The specific role of peroxynitrite in activating RhoA was further examined by comparing active RhoA levels in BAEC cultured in high glucose (25 mM) to cultures treated with exogenous peroxynitrite (100 *μ*M). The results showed that both high glucose and exogenous peroxynitrite can directly activate RhoA in BAEC (Figure 4(b)). The effects of high glucose in inducing active RhoA were blunted by cotreatment of BAEC with FeTPPs (2.5 mM).

### 3.8. Inhibiting Peroxynitrite or RhoA Restored NO Production and eNOS Expression in BAEC

**Figure 5:**
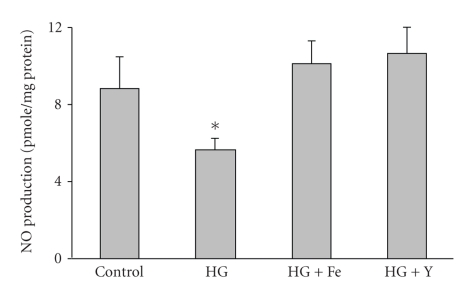
Inhibiting peroxynitrite or Rho kinase restored NO production in BAEC. High glucose (HG, 25 mM) significantly reduced NO production (50%) as measured by NO analyzer in BAEC cultures compared to normal glucose (NG, 5 mM). Cotreatment with FeTPPs (Fe, 2.5 *μ*M) or with the specific Rho-kinase inhibitor Y26732 (Y, 1 *μ*M) restored NO production in high-glucose-maintained cultures. (*n* = 5) Values are expressed as means ± SEM, *P < .05 versus control.

**Figure 6:**
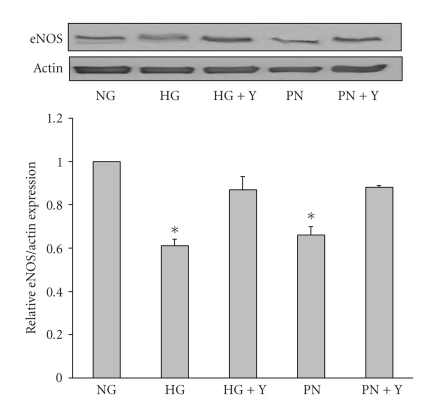
Inhibiting Rho kinase restored eNOS expression in BAEC. Western Blot analysis of eNOS expression showed that cultures of BAEC maintained in high glucose (HG, 25 mM) or peroxynitrite (PN, 100 *μ*M) showed significant reduction in eNOS expression (40%) compared to normal glucose (NG, 5 M). Cotreatment with the specific Rho-kinase inhibitor Y26732 (Y, 1 *μ*M) restored eNOS expression in high glucose or peroxynitrite-maintained cultures. (*n* = 4) Values are expressed as means ± SEM, *P < .05 versus control.

The above results suggest that FeTPPs enhances NO availability via increasing eNOS expression by a mechanism that involves inhibiting RhoA activation. In order to determine the specific contribution of endothelium to the diabetic aortic response, we examined the effects of high glucose on NO production and eNOS expression in BAEC. The results showed that exposure of BAEC to high glucose (25 mM) for 3 days significantly reduced NO production to 64% of basal control levels. Co-treatment with FeTPPs (2.5 *μ*M) or the specific Rho-kinase inhibitor Y26732 (10 *μ*M) restored NO production to control levels (Figure 5). Treatment with Y26732 did not alter the NO production in BAEC cultured in normal glucose (data not shown). We next evaluated the effects on eNOS expression. The results showed that exposure of BAEC to high glucose (25 mM) for 3 days or peroxynitrite (100 *μ*M) for 18 hours significantly reduced eNOS protein expression production to 61% and 66% of basal control levels, respectively. Co-treatment with the specific Rho-kinase inhibitor Y26732 (10 *μ*M) prevented the reduction in eNOS expression and restored it to control levels (Figure 6).

## 4. Discussion

This study has demonstrated that treatment of diabetic rats with FeTPPs, the peroxynitrite decomposition catalyst, for 4 weeks restores diabetes-induced decreases in endothelial-dependent relaxation, reverses the decreased eNOS expression at mRNA levels, and restores NO production in the endothelium of coronary and aortic rat vessels. Similar protective effects of decomposing peroxynitrite were observed in aortic endothelial cell cultures maintained in high glucose. These protective effects of FeTPPs are independent of potential changes in blood glucose concentrations or metabolic alterations related to experimental diabetes including loss of body weight. 

Endothelial dysfunction, as indicated by impaired endothelium-dependent, reflects the inability of the vascular endothelium to generate adequate amounts of NO and to produce NO-mediated relaxation, which has been suggested to be an early event in diabetic atherosclerosis and is associated with coronary artery disease risk (reviewed in [3]). Our previous studies demonstrated that the adverse effects of diabetes on impairing endothelial function were positively correlated with increases in oxidative stress and peroxynitrite formation as indicated by nitrotyrosine [6, 20]. However, the causal role of peroxynitrite in mediating endothelial dysfunction in experimental diabetes remains elusive. This study demonstrates that coronary arteries from STZ-diabetic rats show significant reductions in NO-dependent vasorelaxation in response to Ach, which was significantly restored by treatment of diabetic animals with FeTPPs (15 mg/Kg/day, IP) supporting a key role of peroxynitrite in mediating diabetes-induced endothelial dysfunction. Although endothelial cells from different vascular beds can exhibit metabolic and structural differences [25], diabetes has been reported to impair NO-mediated relaxation in isolated coronary and aortic vessels [6, 11, 20, 26–29]. Increases in oxidative and nitrative stress have been postulated to account for decreases in NO and subsequent formation of peroxynitrite in coronary and aortic vessels [6, 11, 20]. In agreement, our data show that diabetes causes oxidative and nitrative stress in aortic vessels as indicated by increases in lipid peroxidation, a marker for ROS and cell injury as well as nitrotyrosine levels, a marker of peroxynitrite formation. FeTPPs treatment substantially reduced diabetes-induced oxidative and nitrative stress in aortic vessels. 

A considerable body of evidence implicates formation of peroxynitrite as a critical pathogenic element in diabetic endothelial dysfunction. Our finding that FeTPPs improved endothelial-dependent vasorelaxation while reducing oxidative stress prompted us to assess the effect of high glucose on ROS formation by BAEC. Exposure of BAEC to high glucose increased ROS levels as shown by DCF fluorescence, which was blocked by FeTPPs treatment. These data suggest that FeTPPs attenuates diabetes-induced endothelial dysfunction and enhances vascular relaxation by a mechanism involving a decrease in peroxynitrite formation and hence an increase in NO availability. This concept was supported by our finding that FeTPPs prevents high glucose- and diabetes- impaired production of NO in BAEC and aorta homogenate, respectively. Decomposition catalysts, such as FeTPPs and FP15, isomerize peroxynitrite into nitrate [30] and have been proven effective in reducing peroxynitrite-mediated insults in experimental models of diabetes [31–35]. Our results are in good agreement with previous reports showing vascular protective effects of FeTPPs [23, 36–38]. 

To explore another mechanism by which FeTPPs could improve diabetes-induced endothelial dysfunction, we measured eNOS mRNA levels and NO levels in the endothelium of the aorta from various groups. Accumulating evidence indicates that expression of eNOS is regulated by the RhoA/ROCK pathway [14, 15]. Our data showed that diabetes caused significant reductions in eNOS mRNA and NO levels, which were prevented by FeTPPs treatment. Previous reports documented that oxidative stress and diabetes activate RhoA [20, 21, 39, 40]. The likely mechanism by which FeTPPs upregulates eNOS is via reducing ONOO^−^ levels and activation of RhoA and preventing activation of its downstream target Rho kinase, leading to the upregulation of eNOS mRNA expression [14]. This notion is supported by our finding that active RhoA expression has been upregulated in diabetic aorta and in BAEC cultured in high glucose. The protective effects of inhibiting RhoA with FeTPPs were most evident by restoration of NO formation. Thus, upregulation of eNOS may have an important role in the enhanced endothelial-dependent vasorelaxation we observed with FeTPPs treatment. In agreement, recent studies demonstrated reduced expression of eNOS and NO availability in models of diabetes and oxidative stress [11–13]. Of note, our recent studies demonstrated that a parallel mechanism by which activation of RhoA/Rock and subsequent upregulation of arginase expression and activity can contribute to decreases in NO formation and endothelial dysfunction in experimental models of diabetes [20, 41]. 

In summary, our data showed that FeTPPs improves ACh-mediated vasorelaxation, enhances NO formation, and decreases in active RhoA in diabetic rat vessels and high glucose-treated BAEC. We propose that FeTPPs improves diabetes-induced endothelial dysfunction through reducing peroxynitrite formation and restoring eNOS expression and hence increases NO production and availability.

## Abbreviations

U46619: 9,11-Dideoxy-11*α*, 9*α*-epoxymethanoprostaglandin F_2_ *α*
H_2_DCFDA: 2′,7′-dichlorofluorescin diacetate
BAEC: Bovine aortic endothelial cells
ROS: Reactive oxygen species
eNOS: Endothelial NO synthase
K-H buffer: Krebs-Henseleit buffer
HBSS: Hanks' balance salts solution
EBSS: Earle's balanced salt solution.
